# ADSC-Conditioned Medium Mitigates LPS-Induced Acute Lung Injury by Inhibiting Alveolar Macrophage Pyroptosis

**DOI:** 10.3390/cimb48030253

**Published:** 2026-02-26

**Authors:** Fan Yang, Jiachen Li, Ziyi Ren, Chuanyu Zhang, Mingwei Xing, Zhihui Jiao

**Affiliations:** College of Wildlife and Protected Area, Northeast Forestry University, Harbin 150040, China; yangfan0538@nefu.edu.cn (F.Y.);

**Keywords:** acute lung injury, adipose-derived mesenchymal stem cell-conditioned medium, alveolar macrophages, pyroptosis

## Abstract

Acute lung injury (ALI) is characterized by overwhelming pulmonary inflammation and high mortality, yet specific pharmacological interventions remain critically limited. Adipose-derived mesenchymal stem cell-conditioned medium (ADSC-CM) represents a novel cell-free strategy with substantial therapeutic potential. This study investigated the protective effects of ADSC-CM in a rat model of lipopolysaccharide (LPS)-induced ALI. Systemic administration of ADSC-CM significantly attenuated pulmonary pathological damage, reduced systemic inflammatory cytokine levels, and inhibited pyroptosis within lung tissues. Mechanistically, in vitro studies using the NR8383 alveolar macrophage (AM) cell line revealed that ADSC-CM suppressed the TLR4/MyD88/NF-κB signaling axis and the NLRP3/Caspase-1/GSDMD-mediated pyroptotic cascade. These effects were primarily driven by the downregulation of TLR4 expression, although additional molecular targets likely contribute to this protective profile. Our findings highlight the therapeutic efficacy of ADSC-CM in modulating pyroptosis and inflammatory responses in AMs, providing a robust mechanistic rationale for developing ADSC-CM as a cell-free therapeutic platform for the management of ALI.

## 1. Introduction

Acute lung injury (ALI) is a clinical syndrome characterized by distinct pathological alterations in pulmonary tissue architecture, reflecting a systemic inflammatory response localized within the lungs [1]. In clinical veterinary medicine, the substantial costs of treatment combined with a high probability of unfavorable prognoses often necessitate the euthanasia of affected animals, culminating in significant economic losses. Bacterial-induced sepsis is the predominant factor driving ALI progression, with a mortality rate surpassing that associated with other etiologies [2]. At the molecular level, pyroptosis—a pro-inflammatory, regulated form of cell death—is characterized by GSDMD-mediated pore formation, cellular swelling, and rapid lysis [3]. In the context of sepsis-induced ALI, pyroptosis serves a dual function: acting as a defense mechanism against pathogens while potentially exacerbating inflammation and tissue damage when overactivated [4]. Consequently, interventions targeting the pyroptotic pathway may offer a promising therapeutic avenue for ALI [5,6].

As the primary sentinels of the respiratory tract, alveolar macrophages (AMs) represent a critical bridge between pathogen recognition and the initiation of host defense, playing an indispensable role in preserving pulmonary homeostasis [7]. However, in the setting of lipopolysaccharide (LPS)-induced ALI, this defensive posture shifts toward a maladaptive inflammatory response. The activation of the TLR4/NF-κB axis in AMs provides the requisite priming for the assembly of the NLRP3 inflammasome—a multi-protein scaffold where pro Caspase-1 interacts with NLR receptors via the ASC adaptor [8]. The resulting dimerization and autocleavage of pro Caspase-1 yield its catalytically active form, which not only processes the pro-forms of IL-1β and IL-18 into their mature secretable states but also triggers the cleavage of GSDMD [9]. The pore-forming activity of GSDMD eventually culminates in cell pyroptosis, driving the rapid efflux of these potent cytokines into the alveolar space. This process is further compounded by an NF-κB-dependent surge in reactive oxygen species (ROS), which tilts the cellular redox balance toward oxidative stress and compromises organelle integrity [10]. Notably, the release of mitochondrial DNA from dysfunctional mitochondria serves as a secondary molecular trigger, sustaining NLRP3 assembly. This establishes a self-perpetuating cycle of AM pyroptosis, which drives the progressive structural and functional deterioration characteristic of ALI [11,12].

The landscape of regenerative medicine has been fundamentally reshaped by stem cell-based interventions, particularly in addressing complex pathologies where conventional pharmacotherapy often reaches its limits. Beyond the cells themselves, their derivatives have demonstrated a versatile regulatory repertoire—harmonizing immune responses, alleviating oxidative stress, and reinforcing tissue homeostasis. The clinical versatility of mesenchymal stem cell-conditioned medium (MSC-CM) is already evident in its capacity to drive retinal macular repair [13], accelerate the healing of diabetic ulcers [14], and inhibit cancer cell proliferation [15]. This therapeutic potency extends to the pulmonary system, where MSC-CM has been reported to arrest the progression of ALI and preclude its deterioration into acute respiratory distress syndrome (ARDS) [16]. The mechanistic foundation of these effects lies in the stem cell ‘secretome’—a rich molecular payload of growth factors, regulatory peptides, and exosomes liberated via autocrine and paracrine signaling during cellular expansion [17,18,19]. By pivoting to adipose-derived stem cell-conditioned medium (ADSC-CM), researchers can circumvent the inherent safety and logistical bottlenecks of direct cell transplantation while capitalizing on a streamlined, rapid-acting anti-inflammatory and anti-pyroptotic profile. Such a collective ‘cell-free’ strategy offers a sophisticated and clinically viable paradigm for safeguarding pulmonary integrity.

Systemic administration of LPS, particularly via intraperitoneal injection, is a well-validated and robust approach for recapitulating the core pathophysiological hallmarks of human ALI. This model effectively reproduces the clinical “cytokine storm,” [20] characterized by extensive neutrophilic infiltration, alveolar-capillary barrier disruption, and pulmonary edema [21]. Its scientific validity is further underscored by its ability to simulate critical molecular cascades—including TLR4-dependent inflammatory signaling [22] and oxidative stress [23]—that are central to the progression of ALI and ARDS in humans [24]. Consequently, the LPS-induced rodent model provides a high-fidelity preclinical platform for assessing the therapeutic potential of novel interventions, such as ADSC-CM. This approach bridges the gap between experimental mechanistic insights and translational applications, offering significant relevance to both clinical veterinary and human medicine.

In the present study, we first established a Sprague-Dawley (SD) rat model to evaluate the regulatory impact of ADSC-CM on pulmonary inflammation and pyroptosis during LPS-induced ALI. To further elucidate the underlying molecular mechanisms, in vitro experiments were conducted using an AM cell line. By integrating in vivo and in vitro findings, this research aims to provide a robust theoretical and experimental framework for the potential use of ADSC-CM as a novel cell-free therapeutic strategy for ALI.

## 2. Materials and Methods

### 2.1. Isolation, Culture, and Characterization of Adipose-Derived Mesenchymal Stem Cells (ADSCs)

Under sterile conditions, adipose tissue was harvested from the inguinal and axillary fat pads of six-week-old specific-pathogen-free (SPF) female SD rats. All animal experiments adhered to protocols approved by the Laboratory Animal Administration and Ethics Committee of Northeast Forestry University (Approval No. 2022012; 9 March 2022). The excised tissue underwent enzymatic digestion using 0.1% collagenase type I (BioFroxx, Guangzhou, China) at 37 °C for 40 min with continuous agitation. The resulting digested cellular suspension was filtered through a 75 µm mesh to remove residual tissue aggregates. The filtrate was centrifuged at 1500 rpm for 10 min to pellet the cells. Following treatment with red blood cell lysis buffer (Solarbio, Beijing, China) and two subsequent PBS (WILBER, Guangzhou, China) washes, the cell pellet was resuspended for inoculation into T25 culture flasks (Corning, Corning, NY, USA). Cultures of cells were maintained in Dulbecco’s Modified Eagle Medium (DMEM; Gibco, Grand Island, NY, USA), which was enriched with 10% fetal bovine serum (FBS; CAPRICORN, Wuhan, China), 100 U/mL of penicillin, and 100 µg/mL of streptomycin (Solarbio). These were kept in a humidified environment with 5% CO_2_ at 37 °C.

ADSCs at passage four were collected, rinsed with PBS, and stained with fluorochrome-labeled antibodies specific to the rat antigens CD29, CD44h, CD45, and CD90 (BioLegend, San Diego, CA, USA) for 30 min at 4 °C, protected from light. Isotype control antibodies were appropriately employed to determine background fluorescence levels. Following the staining procedure, the cells underwent washing and were analyzed using flow cytometry. To evaluate their multilineage differentiation potential, ADSCs were induced towards adipogenic and osteogenic lineages using specific induction media (Procell, Wuhan, China). The verification of adipogenic differentiation involved Oil Red O staining of intracellular lipid droplets, whereas osteogenic differentiation was assessed by Alizarin Red S staining of calcium deposits.

### 2.2. Generation of Adipose-Derived Mesenchymal Stem Cell-Conditioned Medium (ADSC-CM)

To obtain ADSC-CM, rat ADSCs at passages four were seeded into a T75 culture flask at a concentration of 1 × 10^6^ cells and cultured in DMEM supplemented with 10% FBS, 100 U/mL penicillin, and 100 µg/mL streptomycin until reaching 90% confluence. Following the removal of the culture medium, the cells were washed twice with PBS, and the medium was replaced with 15 mL of serum- and growth factor-free DMEM. The ADSC-CM was collected after a 48-h incubation period and concentrated to approximately 20× using Amicon Ultra-10 Centrifugal Filter Units (Merck Millipore, Billerica, MA, USA). The concentrated ADSC-CM was aliquoted and stored at −80 °C for subsequent use.

### 2.3. Animal Grouping and Treatment

In this study, thirty SPF male SD rats, each with a body weight of 190 ± 20 g, were utilized. The rats were housed under standard laboratory conditions, maintained at a temperature of 24 ± 2 °C with a 12-h light/dark cycle, and were provided with unrestricted access to food and water. The rats were randomly assigned to three experimental groups (*n* = 10 per group) using a random number table. This sample size was determined based on previous literature and the anticipated mortality rate in the LPS-induced model. Specifically: (1) CON group, which received an intraperitoneal injection of 1 mL PBS followed by a tail vein injection of 1 mL DMEM; (2) LPS group, which was administered an intraperitoneal injection of 5 mg/kg LPS (Beyotime, Shanghai, China) and a tail vein injection of 1 mL DMEM; and (3) LPS + CM group, which underwent the same intraperitoneal LPS administration, followed by a tail vein injection of approximately 1 mL ADSC-CM, collected from 1.5 × 10^6^ ADSCs. Following the injections, all animals were maintained under standard laboratory conditions with close clinical monitoring, and no adverse events or signs of distress were observed. Twenty-four hours following the establishment of the model and subsequent treatment, the rats from each group were euthanized under deep isoflurane anesthesia. Immediately thereafter, the thoracic cavity was opened, and blood was collected by cardiac puncture for a complete blood count and serum preparation. Lung tissues were meticulously harvested for further processing and analysis.

### 2.4. ELISA Analysis

Following a two-hour equilibration of rat whole blood samples at ambient temperature, the samples were subjected to centrifugation at 1000× *g* for 20 min to isolate the supernatant. Subsequently, the supernatant was analyzed for IL-1β, IL-6, IL-10, TNF-α, and TGF-β concentrations utilizing ELISA (mlbio, Shanghai, China) in accordance with the manufacturer’s protocol.

### 2.5. Measurement of Lung Wet-to-Dry (W/D) Weight Ratio

To evaluate the extent of pulmonary edema, determine the wet-to-dry (W/D) weight ratio. Following sample collection, utilize absorbent paper to eliminate surface moisture from the lung tissue of the right upper lobe. Immediately thereafter, record the wet weight of the tissue. Subsequently, subject the tissue to a drying process in a controlled environment at 55 °C for a minimum duration of 72 h. Upon completion of the drying period, weigh the tissue again to compute the wet-to-dry weight ratio.

### 2.6. Hematoxylin-Eosin (H&E) Staining and Lung Injury Assessment

Lung tissue blocks were fixed in 4% paraformaldehyde (Saint-Bio, Shanghai, China), embedded in paraffin, and cut into 5 μm thick sections. The sections were subsequently stained with hematoxylin-eosin (H&E) and observed under a light microscope. Lung injury severity was evaluated according to established criteria, including: (1) alveolar congestion, (2) hemorrhage, (3) infiltration of neutrophils into alveolar spaces or vessel walls, and (4) thickening of alveolar walls and/or formation of hyaline membranes. For each section, three non-overlapping fields were randomly selected for evaluation. To ensure objectivity, all slides were coded by a researcher not involved in the study, and the histological scoring was performed by three independent pathologists blinded to the experimental groupings. The cumulative lung injury score was calculated as the sum of the scores from each individual criterion.

### 2.7. Culture and Treatment of NR8383 Cells

The rat AM line NR8383 was purchased from Procell (Wuhan, China). NR8383 cells were seeded at a density of 1.25 × 10^6^ cells per T25 culture flask in 5 mL of NR8383 cell complete medium (Procell) and incubated overnight at 37 °C in a 5% CO_2_ atmosphere. Subsequently, the cells were subjected to the following experimental conditions: (1) CON group: No treatment was applied; (2) LPS group: Cells were treated with 1 μg/mL LPS for 6 h; (3) LPS + CM group: Following a 1-h pre-treatment with 1 μg/mL LPS, cells were exposed to ADSC-CM (collected from 1.25 × 10^6^ ADSCs) and co-cultured for 6 h; (4) LPS + TAK group: Cells were pre-treated with 100 nM TAK-242 (SparkJade, Qingdao, China) for 1 h, followed by stimulation with 1 μg/mL LPS for 6 h; (5) LPS + CM + TAK group: Following a 1-h pre-treatment with 100 nM TAK-242, cells were concurrently treated with 1 μg/mL LPS and ADSC-CM (collected from 1.25 × 10^6^ ADSCs) for 6 h.

### 2.8. Transmission Electron Microscopy (TEM) Observation of AMs Morphology

Lung tissue specimens, approximately 1 mm^3^ in size, or an adequate quantity of NR8383 cells, were washed with PBS and fixed overnight in 2.5% glutaraldehyde (Biosharp, Hefei, China). Following this, the samples were rinsed with 0.1 M phosphate buffer and subsequently fixed with 1% osmium tetroxide for approximately 2 h. After dehydration through a graded series of ethanol and infiltration with acetone, the specimens were embedded in resin. Ultrathin sections of the lung tissue were prepared using an ultramicrotome. These sections or cell samples were then double-stained with 2% uranyl acetate and lead citrate. Finally, images were captured and analyzed utilizing a transmission electron microscope.

### 2.9. Immunofluorescence (IF) Staining and Observation

NR8383 cells were cultured in 12-well plates containing sterile coverslips and subjected to the same treatment groups as previously described. Following fixation with 4% paraformaldehyde, permeabilization with Triton X-100 (Yeasen, Shanghai, China), and washing with PBS, the cells were blocked with 5% BSA for 1 h at room temperature. Subsequently, the cells were incubated overnight at 4 °C with a combination of primary antibodies targeting GSDMD (1:100, Proteintech, 20770-1-AP, Wuhan, China) and NLRP3 (1:100, Proteintech, 68102-1-Ig). After washing with PBS, the cells were incubated for 1 h at room temperature with fluorescent secondary antibodies. Nuclei were stained with the DAPI (Agbio, Changsha, China) working solution for 10 min. Following final PBS washes, images were acquired using a laser scanning confocal microscope.

### 2.10. Western Blot Analysis

Lung tissue homogenates from rats or washed NR8383 cells were lysed using RIPA lysis buffer supplemented with a protease inhibitor cocktail and a phosphatase inhibitor cocktail (Beyotime, Shanghai, China). Following protein concentration quantification via a BCA assay kit (Mei5bio, Beijing, China), equivalent amounts of protein and prestained protein markers (Servicebio, G2091, Wuhan, China) were subjected to electrophoretic separation on 10–15% sodium dodecyl sulfate–polyacrylamide gels. The separated proteins were then transferred onto nitrocellulose membranes with pore sizes of 0.22 μm or 0.45 μm (Biosharp, Hefei, China) at 4 °C. The membranes were blocked with 5% blocking-grade skim milk (BioFroxx, Guangzhou, China) for 1 h, followed by an overnight incubation at 4 °C on a shaker with primary antibodies: TLR4 (1:1000, Abcam, ab22048, Cambridge, MA, USA), MyD88 (1:1000, Wanleibio, WL02494, Shenyang, China), IKKα/β (1:1000, Wanleibio, WL01900), NF-κB (1:2000, Affinity, BF8005, Liyang, China), NLRP3 (1:800, Affinity, BF8029), Pro Caspase-1 (1:3000, Immunoway, YM8437, San Jose, CA, USA), Caspase-1 (1:1500, Wanleibio, WL03450), Pro GSDMD, GSDMD (1:800, Affinity, AF4012), Pro IL-1β, IL-1β (1:3000, Immunoway, YM8498) and IL-18 (1:2000, Proteintech, 60070-1-Ig, Wuhan, China). Following washing with TBST, the membranes were incubated with horseradish peroxidase-conjugated secondary antibodies derived from rabbit (1:5000, Affinity, S0001) or mouse (1:5000, Affinity, S0002) for 1 h at room temperature. Protein bands were subsequently visualized using enhanced chemiluminescence reagents. Finally, protein bands were visualized using enhanced chemiluminescence reagent and imaged with the chemiluminescence imaging system (Servicebio).

### 2.11. Reverse Transcription-Quantitative Polymerase Chain Reaction (RT-qPCR) Analysis

Total RNA was isolated from lung tissue or NR8383 cells utilizing the RNAiso Easy kit (Takara Bio, Beijing, China), following the protocol provided by the manufacturer. For reverse transcription, 1 μg of total RNA served as the template in a 20 μL reaction system, employing the Evo M-MLV RT mix kit. Subsequently, the cDNA was amplified using the SYBR Green Premix Pro Taq HS qPCR kit (Agbio, Changsha, China), along with specific primers synthesized by Genesoul Technology (Harbin, China). The primers employed in this study are listed in Table 1. Gene expression was quantified using the 2^−ΔΔCt^ method and normalized to the expression of GAPDH.

### 2.12. Statistical Analysis

All quantitative data are presented as mean ± standard deviation (SD). Statistical analyses and the generation of graphs were performed using GraphPad Prism 10.6 (San Diego, CA, USA). The normality of data distribution was assessed using the Shapiro–Wilk test prior to analysis. For comparisons among multiple groups, one-way analysis of variance (ANOVA) was employed, followed by Tukey’s post hoc test for multiple comparisons. A *p*-value < 0.05 was considered statistically significant.

## 3. Results

### 3.1. Characterization of ADSCs and Concentration of ADSC-CM

**Figure 1:**
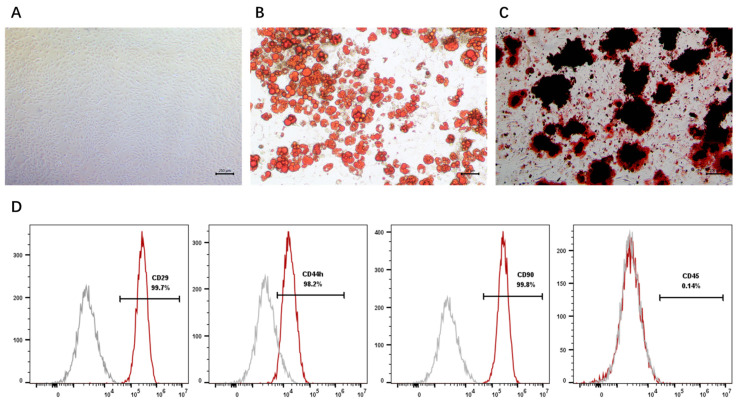
Identification and phenotypic characterization of adipose-derived stem cells (ADSCs). (**A**) Morphological appearance of ADSCs. Scale bar: 250 μm. (**B**) Adipogenic induction and identification of ADSCs. Scale bar: 50 μm. (**C**) Osteogenic induction and identification of ADSCs. Scale bar: 100 μm. (**D**) Flow cytometric analysis of ADSC surface antigens. Vertical and horizontal axes represent fluorescence intensity and cell counts, respectively. The gray curves represent the negative isotype control, and the red curves represent ADSCs stained with specific antibodies for CD29, CD44h, CD90, and CD45.

Primary ADSCs isolated from rat abdominal and inguinal adipose tissues exhibited characteristic spindle-shaped, fibroblast-like morphology and robust plastic adherence under standard culture conditions (Figure 1A). The multipotency of the ADSCs was validated by their successful differentiation into adipogenic and osteogenic lineages, as evidenced by positive Oil Red O (Figure 1B) and Alizarin Red S staining, respectively (Figure 1C). Immunophenotypic characterization via flow cytometry revealed that passage 4 (P4) cells were strongly positive for mesenchymal surface markers CD29 (99.70%), CD44h (98.20%), and CD90 (99.80%), while the expression of the hematopoietic marker CD45 remained negligible (0.14%) (Figure 1D). These findings confirmed the purity and mesenchymal identity of the isolated cell population. Collectively, these findings confirm that the isolated ADSCs maintain both their characteristic immunophenotype and functional multipotency.

For the preparation of the secretome-rich conditioned medium, the starvation medium collected from P4 ADSCs was processed via 10 kDa ultrafiltration, achieving a approximately 15-fold volumetric concentration. The resulting ADSC-CM was immediately cryopreserved at −80 °C to maintain bioactivity for subsequent experiments.

### 3.2. Mitigation of LPS-Induced Systemic Inflammation by ADSC-CM

**Figure 2:**
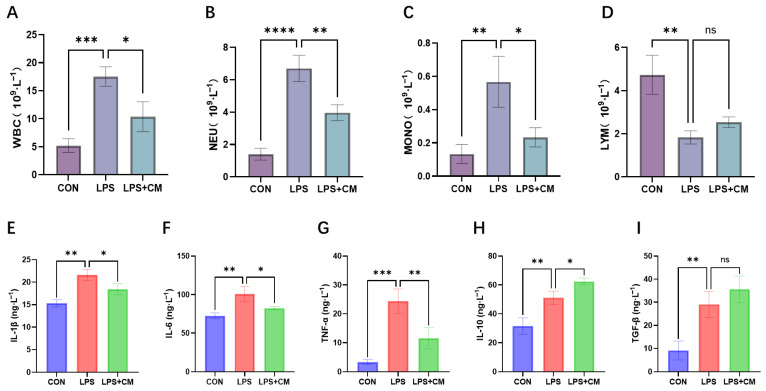
Systemic inflammatory response and hematological parameters in rats. (**A**–**D**) Quantitative analysis of (**A**) WBC, (**B**) NEU, (**C**) MONO, and (**D**) LYM counts in peripheral blood. (**E**–**I**) Serum levels of (**E**) IL-1β, (**F**) IL-6, (**G**) TNF-α, (**H**) IL-10, and (**I**) TGF-β measured by ELISA. Results are expressed as mean ± standard deviation (SD), * *p* < 0.05, ** *p* < 0.01, *** *p* < 0.001, **** *p* < 0.0001; ns, not significant (*p* > 0.05). *n* = 6 biological replicates.

Hematological analysis revealed that LPS challenge induced a profound systemic inflammatory response, characterized by significant leukocytosis—marked by elevated white blood cell (WBC), neutrophil (NEU), and monocyte (MONO) counts (Figure 2A–C)—accompanied by a distinct decrease in lymphocytes (LYM) (Figure 2D). These alterations, indicative of an acute inflammatory state or sepsis-like syndrome, were significantly mitigated following ADSC-CM intervention. Notably, the LPS + CM group exhibited a substantial reduction in total WBC and NEU counts, with concurrent restoration of MONO and LYM levels toward homeostatic ranges.

Consistent with the hematological shifts, serum cytokine profiling via ELISA demonstrated that LPS-induced systemic toxicity was driven by the hyperactivation of pro-inflammatory mediators, including IL-1β, IL-6, and TNF-α (Figure 2E–G). In contrast, systemic administration of ADSC-CM robustly suppressed these pro-inflammatory cytokines while concurrently upregulating the expression of anti-inflammatory factors, specifically IL-10 and TGF-β (Figure 2H–I). Collectively, these findings suggest that tail vein injection of ADSC-CM effectively palliates the excessive systemic inflammatory cascade triggered by LPS, providing a protective buffer against septic injury.

### 3.3. Alleviation of Pulmonary Inflammation by ADSC-CM in ALI Rats

**Figure 3:**
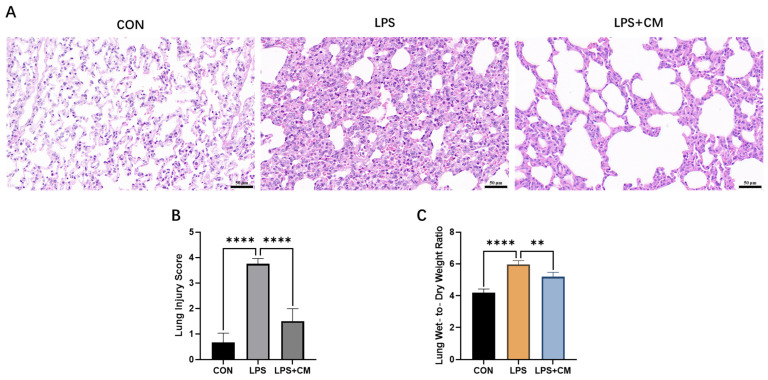
Evaluation of lung injury in rats. (**A**) Representative H&E-stained lung sections. Scale bar: 50 μm. (**B**) Histopathological lung injury scores. (**C**) Lung wet-to-dry (W/D) weight ratio. Results are expressed as mean ± SD, ** *p* < 0.01, **** *p* < 0.0001. *n* = 6 biological replicates.

To assess the severity of pulmonary vascular leakage, the lung W/D weight ratio was determined. The LPS group exhibited a significantly higher W/D ratio compared to the CON group, indicating profound pulmonary edema and impaired alveolar-capillary barrier function. However, this increase was markedly attenuated in the LPS + CM group, suggesting that ADSC-CM intervention effectively palliates fluid extravasation (Figure 3C).

Histopathological evaluation via H&E staining further corroborated these findings. Lung sections from the CON group displayed normal architectural integrity with thin-walled alveoli and no evidence of inflammatory infiltration. In contrast, the LPS-challenged rats exhibited typical hallmarks of ALI, including pronounced alveolar septal thickening, diffuse atelectasis (alveolar collapse), and massive neutrophilic infiltration into the interstitial and alveolar spaces. Additionally, significant intra-alveolar hemorrhage and interstitial edema were observed. Compared to the LPS group, the LPS + CM group exhibited a significant restoration of pulmonary morphology, characterized by a more preserved and intact alveolar architecture (Figure 3A). Semi-quantitative histopathological scoring by independent pathologists confirmed these morphological improvements, showing a significant reduction in injury scores for the LPS + CM group (Figure 3B).

Collectively, these results demonstrate that ADSC-CM treatment effectively rescues the lung from the structural and exudative damage triggered by LPS-induced ALI.

### 3.4. Inhibitory Effects of ADSC-CM on Lung Tissue Pyroptosis

**Figure 4:**
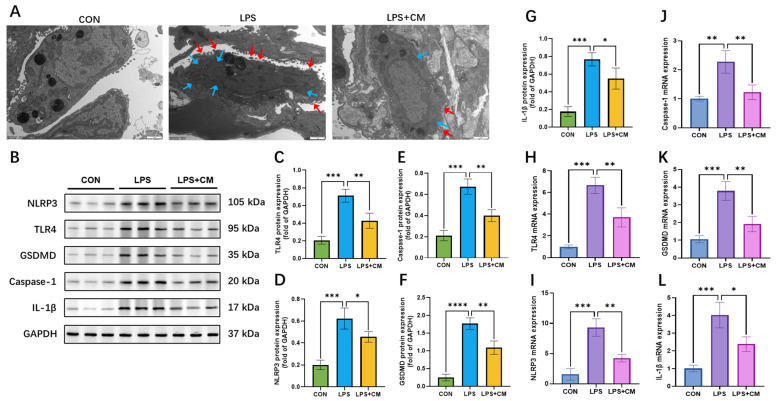
Pulmonary macrophage ultrastructure and pyroptosis marker expression in lung tissues. (**A**) TEM observation of pulmonary macrophages. Red arrow: cell membrane rupture; blue arrow: swollen mitochondria. Scale bars: 1 μm. (**B**–**G**) Western blot analysis: (**B**) Representative bands and protein levels of (**C**) TLR4, (**D**) NLRP3, (**E**) Caspase-1, (**F**) GSDMD, and (**G**) IL-1β. (**H**–**L**) RT-qPCR analysis of mRNA levels for (**H**) *Tlr4*, (**I**) *Nlrp3*, (**J**) *Caspase-1*, (**K**) *Gsdmd*, and **(L**) *Il-1b*. Results are expressed as mean ± SD, * *p* < 0.05, ** *p* < 0.01, *** *p* < 0.001, **** *p* < 0.0001. Protein and mRNA levels were normalized to GAPDH. *n* = 3 biological replicates.

Transmission electron microscopy (TEM) was employed to examine the ultrastructural changes in pulmonary macrophages (Figure 4A). Macrophages from the CON group exhibited intact plasma membranes and well-organized crystalline organelles. Conversely, cells in the LPS group displayed pathognomonic morphological features of pyroptosis, including significant cellular tumefaction and distorted morphology. Notable aberrations included irregular cytoplasmic protrusions and focal membrane rupture (red arrows), alongside pronounced mitochondrial swelling characterized by matrix rarefaction and the loss of cristae (blue arrows). ADSC-CM intervention largely mitigated these ultrastructural injuries, as evidenced by restored membrane continuity and better-preserved mitochondrial architecture.

To further elucidate the molecular mechanisms, the expression of key proteins in the canonical pyroptosis pathway was evaluated (Figure 4B). Western blot analysis revealed that LPS challenge triggered a robust upregulation of TLR4, NLRP3, Caspase-1, GSDMD, and IL-1β in lung tissues. Systemic administration of ADSC-CM, however, significantly suppressed the expression of these pro-pyroptotic mediators (Figure 4C–G). Furthermore, RT-qPCR analysis confirmed that the mRNA levels of these targets were consistent with the protein expression patterns, showing a significant downregulation in the LPS + CM group compared to the LPS-only group (Figure 4H–L).

Collectively, these data indicate that LPS-induced ALI is associated with the activation of TLR4-mediated canonical pyroptosis in the lungs. Notably, ADSC-CM effectively palliates these pathological processes, exerting a protective effect by inhibiting the pyroptotic cascade.

### 3.5. Suppression of LPS-Induced Pyroptosis in NR8383 Macrophages by ADSC-CM

**Figure 5:**
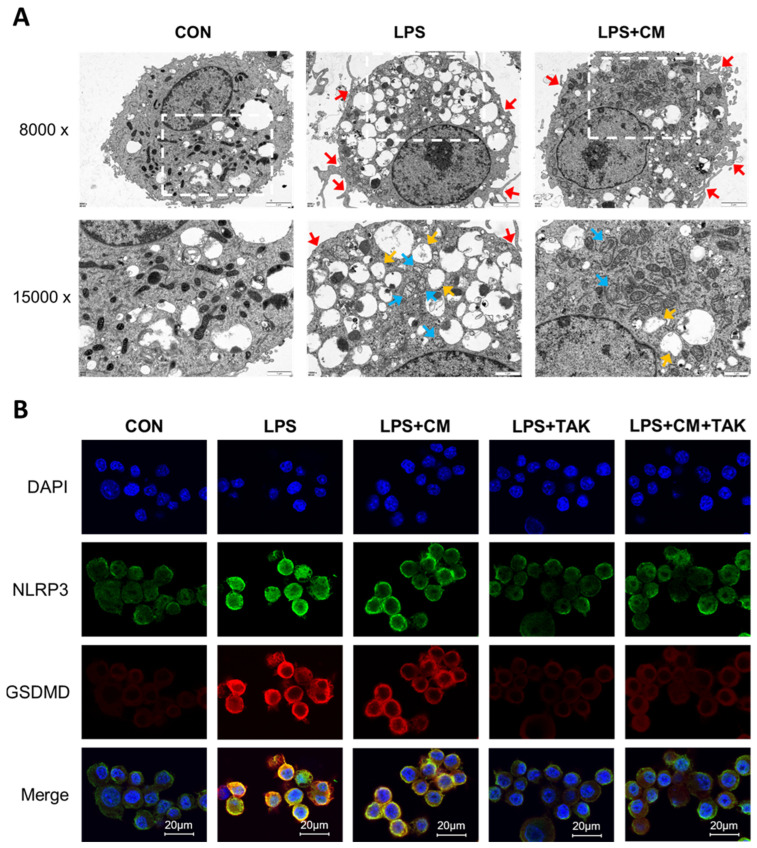
Ultrastructure and immunofluorescence (IF) detection of pyroptosis markers in NR8383 cells. (**A**) TEM observation of NR8383 cells. White dotted boxes in the 8000× images denote the specific regions presented at 15,000× magnification in the panels below. Red arrows: membrane rupture; blue arrows: swollen mitochondria; orange arrows: dilated rough endoplasmic reticulum. Scale bars: 2 μm for low magnification, 1 μm for high magnification. (**B**) IF imaging of NLRP3 and GSDMD. Scale bars: 20 μm.

Ultrastructural analysis via TEM (Figure 5A) revealed that NR8383 cells in the CON group maintained a canonical macrophage morphology with intact plasma membranes. Following LPS challenge, cells exhibited hallmark features of pyroptosis, including marked cellular hypertrophy and the emergence of localized membrane perforations. The cell surface was characterized by numerous irregular cytoplasmic projections and filopodia-like extensions. Notably, the endoplasmic reticulum displayed significant luminal expansion and dilation, progressing into prominent vacuole-like structures. In the LPS + CM group, although some focal membrane discontinuities persisted, the overall cellular architecture was substantially preserved; mitochondria maintained a largely normal morphology, and ER dilation was considerably attenuated compared to the LPS group.

To further characterize these morphological findings, IF staining was performed to visualize the activation of key pyroptotic markers (Figure 5B). Enhanced immunoreactivity for NLRP3 and GSDMD was observed in the LPS group compared to the control group. Conversely, the fluorescence signals for both markers appeared to be attenuated in NR8383 cells co-cultured with ADSC-CM, suggesting a potential inhibitory effect on pyroptosis.

These integrated findings demonstrate that ADSC-CM effectively palliates LPS-induced pyroptosis in NR8383 cells, preserving both membrane integrity and organelle homeostasis.

### 3.6. Regulatory Role of ADSC-CM in TLR4-Mediated Pyroptosis of NR8383 Cells

**Figure 6:**
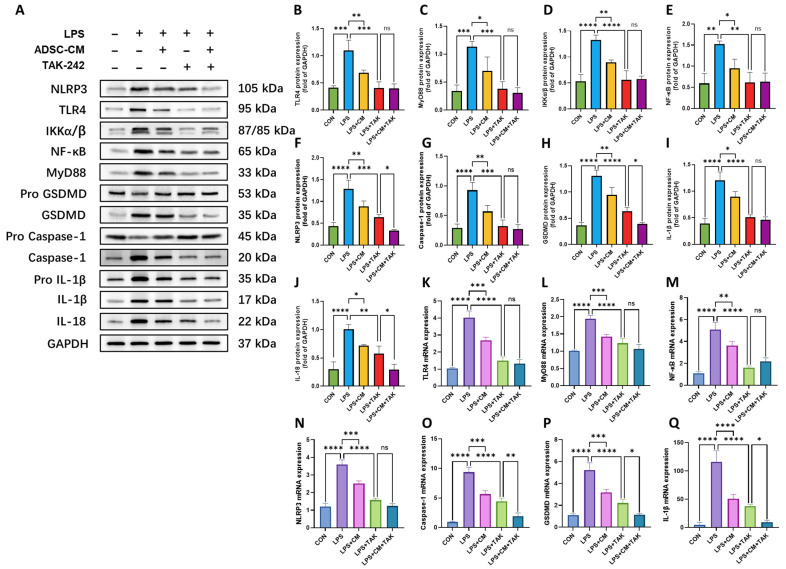
Expression of inflammation and pyroptosis markers in NR8383 cells. (**A**–**J**) Western blot analysis: (**A**) Representative bands and protein levels of (**B**) TLR4, (**C**) MyD88, (**D**) IKKα/β, (**E**) NF-κB, (**F**) NLRP3, (**G**) Caspase-1, (**H**) GSDMD, (**I**) IL-1β, and (**J**) IL-18. (**K**–**Q**) RT-qPCR analysis of mRNA levels for (**K**) *Tlr4*, (**L**) *Myd88*, (**M**) *Nfkb*, (**N**) *Nlrp3*, (**O**) *Caspase-1*, (**P**) *Gsdmd*, and (**Q**) *Il-1b*. Results are expressed as mean ± SD, * *p* < 0.05, ** *p* < 0.01, *** *p* < 0.001, **** *p* < 0.0001; ns, not significant (*p* > 0.05). Protein and mRNA levels were normalized to GAPDH. *n* = 3 biological replicates.

To further investigate the mechanistic basis of ADSC-CM’s protective effects, we analyzed the TLR4/NF-κB inflammatory axis and the NLRP3/IL-1β canonical pyroptosis pathway. Western blot analysis (Figure 6A) demonstrated that LPS challenge triggered a robust upregulation of TLR4, MyD88, IKKα/β, and NF-κB, alongside significant accumulation of NLRP3, Caspase-1, GSDMD, and IL-1β. Notably, both ADSC-CM treatment and pharmacological inhibition of TLR4 (TAK-242) significantly attenuated the expression of these proteins to varying degrees (Figure 6B–J).

Intriguingly, when comparing the LPS + TAK group to the LPS + CM + TAK group, we observed that the addition of ADSC-CM led to a further, significant reduction in the levels of downstream markers—including NLRP3, GSDMD, and IL-18—whereas the expression of upstream components such as TLR4, MyD88, and NF-κB showed no statistically significant difference between these two groups. Parallel RT-qPCR analysis confirmed that the mRNA expression patterns of these targets were generally consistent with the protein levels (Figure 6K–Q), reinforcing the observation that ADSC-CM effectively suppresses the LPS-induced activation of the TLR4-mediated inflammatory and pyroptotic pathways.

In summary, these findings demonstrate that LPS challenge activates the canonical TLR4-mediated pyroptotic pathway in NR8383 cells, significantly upregulating pro-inflammatory factors and hallmark pyroptotic indicators. Treatment with ADSC-CM markedly inhibits this pyroptotic response. Within this therapeutic process, TLR4 serves as a primary regulatory target for ADSC-CM to modulate AM inflammation and pyroptosis; meanwhile, additional potential regulatory targets within the NLRP3/IL-18 signaling cascade are also involved in the protective effects of ADSC-CM.

## 4. Discussion

ALI is characterized by multifactorial pathogenesis, rapid clinical progression, and high mortality rates, yet specific pharmacological interventions remain elusive in clinical practice. Recently, the therapeutic potential of mesenchymal stem cells (MSCs) and their derivatives has gained significant attention. For instance, bone marrow-derived MSCs (BMSCs) were shown to mitigate bleomycin-induced pulmonary fibrosis as early as 2003 [25]. However, direct MSC transplantation entails inherent risks, including immunological rejection, embolic events, pro-fibrotic potential, and tumorigenicity [26]. Consequently, stem cell-CM has emerged as a promising cell-free alternative, offering distinct advantages such as ease of preparation, administrative flexibility, superior tissue penetration, long-term stability, and an enhanced safety profile [27].

As a prototypical endotoxin derived from Gram-negative bacteria, LPS remains a canonical tool for recapitulating inflammatory landscapes across diverse organs, most notably the lungs and kidneys. Its capacity to trigger TLR4 signaling is indispensable for probing the molecular mechanisms that underpin sepsis-related injury [28,29,30]. Within the pulmonary immune microenvironment, macrophages function as central orchestrators; consequently, modulating their polarization states or pyroptotic pathways has emerged as a high-value strategy for tempering the hyper-inflammatory response characteristic of ALI [31,32,33]. AMs, in particular, are uniquely positioned as ‘sentinel cells’—being the first population to undergo pyroptosis and subsequently driving the inflammatory crosstalk within lung parenchyma [2]. This early-stage pyroptotic event in AMs acts as a molecular initiator, triggering a self-amplifying cascade that directly exacerbates the structural and functional deterioration seen in LPS-induced ALI [34].

In our animal model, a 24-h intraperitoneal LPS challenge successfully established a robust systemic inflammatory milieu, characterized by a profound surge in serum pro-inflammatory mediators (IL-1β, IL-6, and TNF-α). This systemic insult translated into severe pulmonary architectural disruption, as evidenced by the elevated lung W/D weight ratio and characteristic histopathological lesions observed in H&E-stained sections, consistent with prior reports [35,36]. Critically, ultrastructural profiling via TEM provided direct morphological evidence of pyroptosis within alveolar macrophages, where classic hallmarks—including membrane poration, organelle swelling, and cristae fragmentation—were prevalent. This pyroptotic phenotype was further corroborated at the molecular level by the concerted activation of the TLR4/NLRP3/Caspase-1 signaling axis. The accompanying elevation of GSDMD-NT—the executive protein of pore formation—alongside the maturation and release of cleaved IL-1β, explicitly confirms that the LPS-induced pulmonary insult is underpinned by a hyperactive pyroptotic cascade. In contrast to the LPS group, systemic administration of ADSC-CM through the tail vein significantly reversed the progression of ALI, as evidenced by the synchronized downregulation of key pyroptotic markers at both the protein and mRNA levels. While previous literature has underscored the potential of human umbilical cord- or adipose-derived MSC exosomes in mitigating macrophage pyroptosis in murine models [34,37], the present study expands this paradigm. To our knowledge, this is the first study to demonstrate the therapeutic efficacy of rat-derived ADSC-CM in a homologous rat model of ALI via intravenous delivery. These findings not only validate the anti-pyroptotic properties of ADSC-CM but also highlight its potential as a cell-free therapeutic strategy for acute respiratory distress.

Moreover, we observed that the protein and mRNA expression levels of TLR4 in the lung tissues of rats in the LPS + CM group were significantly lower than those in the LPS group. Previous studies have identified TLR4 as a type I transmembrane glycoprotein characterized by a horseshoe structure [38], which can form heterodimers with the accessory protein MD-2 [39]. In the presence of LPS, LPS and lipopolysaccharide binding protein (LBP) are presented to the hydrophobic pocket of MD-2 by CD14 [40], inducing conformational changes in MD-2. Under the influence of electrostatic and symmetric interactions, the (TLR4/MD-2/LPS)2 dimer structure, capable of activating TLR4, is ultimately formed [41]. With its unique structure and flexible assembly mechanism, TLR4 efficiently recognizes and signals LPS. By inhibiting TLR4 expression, the release of inflammatory factors and the occurrence of pyroptosis can be effectively suppressed [22,42]. Relevant studies have confirmed that microRNA (miR)-515-5p, contained in bone marrow mesenchymal stem cell exosomes (BMSC-Exo), can directly target TLR4 to inhibit NLRP3 inflammasome-mediated pyroptosis and inflammation, while enhancing the antioxidant stress capacity of mitochondria, thereby providing a protective effect on the nerve and bone tissues of rats [43,44]. Consequently, we predict that TLR4 is one of the regulatory sites of ADSC-CM on inflammation and pyroptosis in AMs, and we will conduct subsequent cellular experiments.

For our cellular experiments, we selected the rat alveolar macrophage cell line NR8383 as the research subject and introduced resatorvid (TAK-242) to conduct an in-depth investigation of the underlying mechanisms. The NR8383 cell line is widely recognized as an effective experimental model for in vitro studies of LPS-induced pulmonary inflammation [45]. TAK-242 serves as a selective inhibitor of TLR4, effectively blocking the activation of both MyD88-dependent and TRIF-dependent signaling pathways by targeting the binding of the Cys747 residue in the TLR4 domain to the TIRAP and TRAM proteins [46,47].

In our in vitro cellular experiments, the optimal LPS concentration for modeling was determined to be 1 μg/mL by integrating insights from published literature with our internal pilot study results [48,49]. Additionally, it was demonstrated that ADSC-CM exhibited no cytotoxicity towards NR8383 cells, a finding that aligns with previous studies [50]. The visual evidence obtained through TEM and IF staining offered a clear contrast: LPS-induced pyroptotic features were visibly suppressed in cells co-cultured with ADSC-CM, consistent with our molecular findings. At the molecular level, ADSC-CM markedly suppressed the TLR4-primed MyD88/NF-κB axis and the subsequent NLRP3/Caspase-1/GSDMD signaling cascade, underscoring its dual role in dampening both inflammatory priming and pyroptotic execution.

Notably, MyD88 serves as a seminal adapter protein that initiates the transduction of extracellular signals into intracellular cascades, bridging the initial inflammatory environment of ALI to the terminal execution of AM pyroptosis. Recent evidence indicates that whether via S100A9 gene deletion [51] or the application of Chlojaponilactone B [52], the fundamental pathway for mitigating macrophage pyroptosis centers on intercepting this MyD88-dependent TLR4/NF-κB axis. Beyond its role as a linear transducer, MyD88 facilitates a self-amplifying “pyroptotic loop.” As documented by He et al., the activation of the MyD88/NF-κB pathway upon LPS-TLR4 binding stimulates the expression of IL-1 receptor I (IL-1RI) on the AM surface. This metabolic priming renders the cells hyper-sensitive to IL-1β, creating a feedback mechanism that intensifies macrophage lysis and drives the pathological progression of ALI [53]. Our data demonstrate that ADSC-CM effectively breaks this vicious cycle by suppressing the MyD88/NF-κB axis [54]. This multifaceted regulatory profile highlights why ADSC-CM provides more robust systemic protection than interventions restricted to a single molecular target.

The molecular mechanisms underlying the protective effects of ADSC-CM were further elucidated through the selective inhibition of TLR4 using TAK-242. In our experiments, the absence of statistically significant differences in MyD88, IKKα/β, and NF-κB protein and mRNA expression between the LPS + TAK and LPS + CM + TAK groups confirms that ADSC-CM utilizes TLR4 as a primary upstream intervention site. Notably, however, our molecular detection revealed that the levels of downstream pyroptotic indicators—including NLRP3 and GSDMD—were significantly lower in the LPS + CM + TAK group compared to the LPS + TAK group. This divergence indicates that the anti-pyroptotic efficacy of ADSC-CM is not restricted to the TLR4/MyD88 interface alone; it likely extends to the modulation of additional, TLR4-independent pathways within the pyroptotic cascade. Such a multi-layered regulatory profile allows ADSC-CM to more effectively address the multifaceted inflammatory response in alveolar macrophages than single-target inhibition.

Previous research into the molecular components of MSC derivatives provides a theoretical basis for the multi-targeted protection observed with ADSC-CM. For instance, miR-378a-5p found in MSC exosomes has been identified to possess direct binding sites for NLRP3, effectively antagonizing inflammasome activation and alleviating pyroptosis-mediated inflammation [55]. Extending this paradigm, Yue et al. confirmed that miR-182-5p, a key cargo identified in mouse BMSC exosomes, can specifically target GSDMD and significantly reduce pyroptosis in cardiomyocytes [56]. The regulatory depth of these derivatives is further exemplified by the work of Mao et al., which demonstrated that LncRNA KLF3-AS1 can modulate the miR-138-5p/SIRT1 axis to enhance the inhibitory effect on NLRP3 [57]. Furthermore, Liu et al. showed that miR-16-5p directly downregulates Caspase-1 expression, thereby suppressing the pyroptotic cascade through various immunomodulatory proteins [34]. Beyond direct targeting, MSC-derived extracellular vesicles have been shown to inhibit the NOX4/ROS pathway and activate the Nrf2 antioxidant system via specific miRNAs, effectively mitigating mitochondrial oxidative damage and subsequent cell death [58]. Notably, in models of chronic inflammatory pain, MSC-derived exosomes have even been found to enhance the synthesis of autophagy-related proteins through the miR-146a-5p/TRAF6 axis, facilitating the degradation of the ASC adapter to prevent pro-IL-1β maturation [55]. While individual studies often focus on specific exosomal cargos, our data suggest that the cumulative effect of ADSC-CM is likely much broader. The capacity of the ADSC-CM secretome to integrate macrophage autophagy, oxidative stress, and the suppression of core executors like NLRP3 and GSDMD hints at a systemic protective influence on AMs. Such multifaceted bioactivity—remarkable for its mechanistic depth and its capacity to fundamentally preserve cellular homeostasis—provides a compelling rationale for more granular exploration in future research.

## 5. Conclusions

In summary, ADSC-CM represents a promising cell-free therapeutic strategy that effectively attenuates pulmonary inflammation and suppresses AMs pyroptosis in ALI models. Mechanistically, our findings identify the TLR4 signaling pathway as a primary regulatory target of ADSC-CM in modulating pyroptotic cell death, although the involvement of additional molecular pathways remains to be elucidated. Despite these insights, the specific bioactive components within ADSC-CM responsible for this therapeutic efficacy have yet to be fully characterized. Furthermore, while the current rat model successfully recapitulates acute injury, it may not encompass the long-term clinical progression of the disease. Future research should prioritize identifying the key functional molecules through proteomic or exosomal analysis and validating these findings in clinically relevant large-animal models. Exploring optimized delivery routes, such as nebulization, will be essential to fully realize the therapeutic potential of ADSC-CM and advance clinical interventions for ALI.

## Figures and Tables

**Table 1:** Information of primers employed for RT-qPCR.

Primer Name	Forward	Reverse
TLR4	5′-CCGCTTCCAGATCGTACAACT-3′	5′-AGACTCCTATCTGCCTCACT-3′
MyD88	5′-CTCCAGGTGTCCAACAGAAG-3′	5′-TGGTATAGTCGCAGATAGTGATGA-3′
NF-κB	5′-AGGACTGCCGGGATGGCTTCTAT-3′	5′-GGTCTGGATGCGCTGGCTAATGG-3′
NLRP3	5′-CAGAAGCTGGGGTTGGTGAA-3′	5′-CCCATGTCTCCAAGGGCATT-3′
Caspase-1	5′-GACCGAGTGGTTCCCTCAAG-3′	5′-GACGTGTACGAGTGGGTGTT-3′
GSDMD	5′-AAGATCGTGGATCATGCCGT-3′	5′-CGGGGTTTCCAGAACCATGA-3′
IL-1β	5′-CAGCTTTCGACAGTGAGGAGA-3′	5′-TTGTCGAGATGCTGCTGTGA-3′
GAPDH	5′-ACTTTGGCATCGTGGAAGGG-3′	5′-ACATTGGGGGTAGGAACACG-3′

## Data Availability

The original contributions presented in this study are included in the article. Further inquiries can be directed to the corresponding authors.

## Abbreviations

The following abbreviations are used in this manuscript:

ADSC-CM	Adipose-derived mesenchymal stem cell-conditioned medium
ALI	Acute lung injury
AMs	Alveolar macrophages
